# Dental Mesenchymal Stem Cell Secretome: An Intriguing Approach for Neuroprotection and Neuroregeneration

**DOI:** 10.3390/ijms23010456

**Published:** 2021-12-31

**Authors:** Agnese Gugliandolo, Emanuela Mazzon

**Affiliations:** IRCCS Centro Neurolesi “Bonino-Pulejo”, Via Provinciale Palermo, Contrada Casazza, 98124 Messina, Italy; agnese.gugliandolo@irccsme.it

**Keywords:** dental mesenchymal stem cells, secretome, conditioned medium, extracellular vesicles, exosome, neuroregeneration, neuroprotection, neuronal differentiation

## Abstract

Mesenchymal stem cells (MSCs) are known for their beneficial effects and regenerative potential. In particular, dental-derived MSCs have the advantage of easier accessibility and a non-invasive isolation method. Moreover, thanks to their neural crest origin, dental MSCs seem to have a more prominent neuroregenerative potential. Indeed, in basal conditions they also express neuronal markers. However, it is now well known that the beneficial actions of MSCs depend, at least in part, on their secretome, referring to all the bioactive molecules released in the conditioned medium (CM) or in extracellular vesicles (EVs). In this review we focus on the applications of the secretome derived from dental MSCs for neuroregeneration and neuroprotection. The secretomes of different dental MSCs have been tested for their effects for neuroregenerative purposes, and the secretomes of dental pulp stem cells and stem cells from human exfoliated deciduous teeth are the most studied. Both the CM and EVs obtained from dental MSCs showed that they are able to promote neurite outgrowth and neuroprotective effects. Interestingly, dental-derived MSC secretome showed stronger neuroregenerative and neuroprotective effects compared to that obtained from other MSC sources. For these reasons, the secretome obtained from dental MSCs may represent a promising approach for neuroprotective treatments.

## 1. Introduction

Mesenchymal stem cells (MSCs) are multipotent cells with great potential for regenerative medicine [1]. MSCs were first isolated in the bone marrow by Friedenstein et al. [2,3]. However, the term MSCs was coined later by Caplan, indicating their multipotent differentiation ability to give rise to mesodermal lineage [4]. In 2006, Dominici et al. established the criteria to classify MSCs, which are the plastic adherence ability in standard culture conditions, the expression of CD105, CD73 and CD90, the lack of CD45, CD34, CD14 or CD11b, CD79alpha or CD19 and HLA-DR surface molecules, and the differentiation potential toward osteoblasts, adipocytes and chondroblasts in vitro [5].

Since the first discovery, MSCs have been isolated from different tissues. Regarding dental tissues, in 2000 Gronthos et al. firstly isolated a population of MSCs from dental pulp cells, with similar properties to bone marrow MSCs (BMSCs) [6]. Since then, different dental-derived cells have been found to possess stem cell properties and were named according to their tissue of origin, including dental pulp stem cells (DPSCs), stem cells from human exfoliated deciduous teeth (SHEDs), periodontal ligament stem cells (PDLSCs), dental follicle stem cells (DFSCs), stem cells from apical papilla (SCAPs), and gingival MSCs (GMSCs) [7].

Dental MSCs have the advantages of being easily accessible with minimally invasive procedures [8], expandable with relative genomic stability for a long period of time, and show immunomodulatory properties [9]. Moreover, they are also able to differentiate toward the mesodermal lineage, but they also show the ability to transdifferentiate into ectodermal and endodermal lineages [10].

Dental MSCs have neural crest origin and for this reason they show more potent neurogenic capabilities compared to other MSCs [11]. Thanks to their origin, dental MSCs express some neural progenitor and mature cell markers, even when not exposed to neural induction medium and in standard culture conditions, such as nestin, β-3 tubulin, neurotrophin receptors, and neurofilaments [12,13]. In addition, dental MSCs show a greater differentiation potential for neurogenesis compared to other MSCs types [14,15]. Thus, dental MSCs, thanks to their differentiation potential and paracrine effects, may represent a good source of MSCs for the treatment of neurodegenerative disorders and for neural regeneration [16,17,18,19,20].

The beneficial properties of MSCs are often associated with their differentiation potential. Indeed, MSCs differentiating toward neuronal cells may replace degenerated ones. However, it is now well accepted that MSCs’ regenerative and protective effects are mediated also by their secretome. In this review, we focus on the secretome obtained by dental MSCs, showing its potential for neuroprotection and neuroregeneration in preclinical models.

## 2. MSCs Secretome

The MSC secretome includes various bioactive molecules, such as lipids, proteins, nucleic acid, chemokines, cytokines, growth factors, and hormones, released in their conditioned medium (CM) or extracellular vesicles (EVs) [21].

The application of the secretome for cell-free therapy seems promising and has the advantage of not having the ethical limits related to the use of stem cells, and shows low immunogenicity [22]. In addition, some reports indicate only a limited survival of MSCs after transplantation [23].

EVs may also play central roles in cell-free therapies. EVs are membrane-bound bilayered lipid particles, secreted by different cell types, carrying a cargo of biological molecules from their parent cells. They are important mediators of biological information in intercellular cell signaling from the parent into a recipient cell. EVs are classified as microvesicles (MVs), exosomes (EXOs), and apoptotic bodies on the basis of their size but also of other features such as biogenesis and release pathways [24,25]. MVs are produced through direct budding from the cell plasma membrane. On the contrary, EXOs are smaller and originate by an inward budding of the limiting membrane of early endosomes, which mature into multivesicular bodies during the process. After the fusion with the plasma membrane, multivesicular bodies release EXOs into the extracellular milieu [24,26]. EVs, thanks to their surface molecules, can target the recipient cells. Once attached to a target cell, EVs can promote signaling via receptor–ligand interactions or can be internalized by endocytosis, phagocytosis or fuse with the target cell’s membrane and release their content into the cytoplasm [27,28]. EVs released by MSCs contain proteins, lipids, mRNA, microRNA (miRNA), and cytokines. These vesicles release their contents into target cells, modulating their activity and potentially inducing restorative processes [29].

### Dental MSCs Secretome

Interestingly, the secretome profile may be influenced by different MSC sources [30]. For this reason, dental MSCs may present differences in secretome composition compared to other MSCs.

The analysis of SCAPs secretome has evidenced a total of 2046 proteins including chemokines, angiogenic, immunomodulatory, antiapoptotic, and neuroprotective factors other than extracellular matrix (ECM) proteins. Interestingly, the levels of 151 proteins were different by at least twofold compared to BMSCs. Indeed, SCAPs showed increased levels of proteins involved in metabolic processes, transcription, chemokines and neurotrophins while they presented a reduction of those associated with biological adhesion, developmental processes, immune function, ECM proteins and proangiogenic factors [31].

The DPSC secretome contains different cytokines, chemokines and growth factors, including vascular endothelial growth factor (VEGF)-A and Follistatin (FST) that are the most prominent [32]. Another study evidenced that granulocyte colony-stimulating factor (G-CSF)-mobilized DPSCs expressed higher levels of angiogenic and neurotrophic factors, including granulocyte macrophage colony-stimulating factor (GM-CSF), matrix metalloproteinase (MMP) 3, VEGF, and nerve growth factor (NGF) compared to BMSCs and adipose-tissue-derived MSCs (AMSCs). In particular, DPSCs-CM induced greater neurite outgrowth in human neuroblastoma TGW cells. Trophic effects of DPSCs on migration and apoptosis were higher compared to those of BMSCs and AMSCs [33]. The expression levels of cytokines in DPSCs were also compared with developing apical complex cells (DACCs). A total of 25 cytokines were identified, of which 22 were expressed more strongly in DPSCs-CM. Specifically, odontoblast differentiation-related cytokines and the neurotrophin (NT)-3 and NT-4 were more strongly expressed in DPSCs-CM [34]. The protein content of PDLSC-CM was also analyzed by liquid chromatography–tandem mass spectrometry (LC/MS/MS), that detected a total of 99 proteins, including matrix proteins, enzymes, growth factors, cytokines, and angiogenic factors [35]. LC-MS/MS also evidenced the presence of osteogenic proteins in the dental MSCs secretome [36].

Comparative secretome profiling showed the presence of fibroblast growth factor (FGF)-2, interleukin (IL)-10, platelet-derived growth factor (PDGF), stromal cell–derived factor (SDF)-1, angiopoietin (Ang)-1, transforming growth factor (TGF)-β3, hepatocyte growth factor (HGF), interferon (IFN)-γ, VEGF, and IL-6 in CM from SHEDs, BMSCs and Wharton’s-Jelly-derived MSCs (WJMSCs). PDGF-A, IL-10, FGF-2, and SDF-1 were similar in all samples, TGF-β3 and Ang-1 were higher in BMSCs, while HGF and INF-γ showed an increase in SHED. VEGF was increased in WJMSCs [37].

The differences in the secretory factors of human permanent- and deciduous-teeth PDLSCs have also been evaluated. Proteins involved in cell growth, cell communication, and signal transduction were more frequently found in permanent-teeth PDLSCs-CM, together with higher levels of NT-3 and NT-4 and angiogenesis-related cytokines such as epidermal growth factor (EGF) and insulin-like growth factor (IGF)-1. On the contrary, CM obtained from deciduous-teeth PDLSCs contained mainly proteins involved in regulation of the cell cycle and the levels of cytokines involved in immune response and in tissue degradation and catalytic activities, including MMP1, Proteasome subunit, alpha type, 1 (PSMA1), and cullin 7 (CUL7) were higher in these cells [38].

Pulp CD31^−^ side population (SP) cells expressed the highest levels of angiogenic and neurotrophic factors compared to those isolated from bone marrow and adipose tissue. CM from pulp CD31^−^ SP cells showed anti-apoptotic and neurite outgrowth ability [39].

EXOs derived from DPSCs showed stronger immune-modulating capacity compared to BMSCs EXOs. Specifically, DPSCs EXOs inhibited CD4+ T cell differentiation into T helper 17 cells and reduced the secretion of pro-inflammatory cytokines IL-17 and tumor necrosis factor (TNF)-α, while promoting the polarization of CD4+ T cells into T reg and increasing the release of the anti-inflammatory factors IL-10 and TGF-β [40].

The transcripts present in EVs were studied also. GMSCs EVs contained transcripts encoding for several growth factors such as TGF-β, FGF, and VEGF, but also glial-cell-derived neurotrophic factor (GDNF) family ligands and neurotrophins, such as NGF, brain-derived neurotrophic factor (BDNF), NT-3 and NT-4 involved in neuronal development. Some ILs and members of the Wnt family were also present [41].

EVs also contain non-coding RNA. EVs of PDLSCs highlighted the presence of different classes of non-coding RNAs, including antisense RNA and long non-coding RNA (lncRNAs), but also five miRNAs, which are MIR24-2, MIR142, MIR335, MIR490, and MIR296. These miRNAs target genes belonged to the gene ontology class “Ras protein signal transduction” and “Actin/microtubule cytoskeleton organization” [42].

A total of 593 and 920 known PIWI-interacting RNAs (piRNAs) were identified from SCAP-EXOs and BMSC-EXOs, respectively, and 21 piRNAs were differentially expressed. The target genes of the differentially expressed piRNAs were mainly involved in biological regulation, cellular processes, metabolic processes, binding, and catalytic activity. Specifically, the target genes of the upregulated piRNAs in SCAP-EXOs were enriched in the mitogen-activated protein kinase (MAPK) signaling pathway, Ras signaling pathway, and citrate cycle signaling pathway. On the contrary, the target genes of downregulated piRNAs in SCAP-EXOs were enriched in the p53 signaling pathway and Epstein–Barr virus infection signaling pathway [43].

It is important to notice that donor age and microenvironmental conditions in vitro may also influence secretome composition. DPSC-CM obtained in normoxic conditions was reported to be enriched in molecules with anti-inflammatory, tissue repair, and regenerative properties compared to CM obtained in hypoxic conditions [44]. Moreover, secretome collected from 5% O_2_ cultured DPSCs showed higher stimulatory effects on proliferation and migration of mouse embryonic fibroblast NIH3T3 cells and on neuronal differentiation of SH-SY5Y cells [45]. The quantity and size of EXOs and their tetraspanins expression may vary dependent on the medium used for culture [46]. The secretome of SHEDs and young DPSCs contained more growth factors and lower levels of pro-inflammatory cytokines compared to DPSCs obtained from old subjects. Differentiation potential was also higher in SHEDs and young DPSCs [47].

CM can be obtained by healthy PDLSCs but also by inflamed PDLSCs. CM obtained by inflamed ones increased proliferation of both healthy and inflamed PDLSCs, but reduced the differentiation toward osteoblasts. Healthy CM rescued the impaired osteogenic differentiation [48].

The treatment with different substances may also influence cell secretome. The treatment of the DPSCs with 2,3,5,4′-tetrahydroxystilbene-2-O-β-D-glucoside (THSG), a bioactive component of *Polygonum multiflorum* Thunb., induced changes in the secretion of growth-associated proteins in CM, increasing some of them such as AKT2 and NGF receptor [49]. Instead, CM from FGF-2-modified GMSCs contained more VEGF-A, FGF-2, and TGF-β [50]. Ascorbic acid treatment of SHEDs increased the release of growth factors necessary for the tissue regeneration and homeostasis, including VEGF, SCF, IGF-1, HGF, bFGF, Ang-1, and EGF, and anti-inflammatory cytokines, such as NO, indoleamine 2,3-dioxygenase (IDO), PGE-2, IL-10, and IL-6. On the contrary, inflammatory cytokines CCL2 and TGF-β1 were reduced [51].

The exposure to differentiation medium also could induce changes in non-coding RNA in EVs and EXOs of PDLSCs. Specifically, 69–557 circular RNA (circRNAs) and 2907–11,581 lncRNAs were found in EVs isolated from PDLSCs and PDLSCs exposed to osteogenic differentiation medium at different time points. Compared with undifferentiated PDLSCs EVs, 3 circRNAs and 2 lncRNAs were upregulated and 39 circRNAs and 5 lncRNAs were downregulated consistently after 5 and 7 days of exposure to differentiation medium [52]. Moreover, 72 miRNAs were upregulated while 35 were downregulated in PDLSCs EXOs after osteogenic induction [53].

A summary of the main factors found in the secretome of the different dental MSCs can be found in Table 1.

## 3. Dental Stem Cell Secretome Neuroprotective and Neuroregenerative Potential in Preclinical Models

In order to evaluate the neuroregenerative and neuroprotective potential of the dental MSC secretome, the effects of CM and EVs have been evaluated in preclinical models of neurodegenerative and neurological diseases and models of neuronal damage, such as spinal cord injury (SCI). In addition, secretome mediated effects on neuronal outgrowth, its capacity to stimulate neuronal differentiation, and the effects on glial cells have also been evaluated. We performed a PubMed search looking for studies showing the neuroregenerative and neuroprotective potential of dental MSCs secretome in in vitro and in vivo models.

### 3.1. Dental Pulp Stem Cell Secretome

DPSCs secretome was one of the most studied. Different studies evaluated its efficacy in inducing neurite outgrowth. It was reported that DPSC-CM promoted neurite outgrowth in dorsal root ganglion (DRG) neurons. Specifically, the total length and joint number of neurites increased after treatment with CM. Moreover, DPSC-CM promotes Schwann cell viability and myelin formation [54].

DPSCs-CM enhanced cell survival and induced neurite outgrowth of PC12 cells, as shown by neuronal nuclear protein (NeuN), microtubule-associated protein 2 (MAP-2) and βIII-tubulin. Specifically, DPSCs-CM was more efficacious in inducing PC12 neurite outgrowth compared to DPSCs/PC12 co-cultures, indicating that cells co-cultures had a delayed lag time in producing efficacious amounts of trophic factors. DPSCs-CM also enhanced cell migration. Interestingly, the number of surviving PC12 cells was reduced when CM was added with anti-GDNF. Instead, the addition of anti-NGF, anti-GDNF and anti-BDNF antibodies attenuate PC12 neurite outgrowth. These data demonstrated that NGF, BDNF and GDNF are involved in the PC12 survival and differentiation [55].

The DPSC secretome shows a chemoattractive effect on SH-SY5Y cells. Moreover, its effect on neural maturation has been evaluated. With this aim, SH-SY5Y cells were induced toward neuronal cells and after they were exposed to the DPSC secretome. SH-SY5Y cells subjected to the DPSC secretome showed increased neurite outgrowth, acquired ultrastructural features of neuronal cells and presented an increased immune reactivity for neuronal markers. Moreover, CM-treated SH-SY5Y cells developed distinct features including Cd2+-sensitive currents, which suggests that CM-DPSC-maturated SH-SY5Y acquired voltage-gated Ca^2+^ channels [56]. In line with the previous study, CM obtained by DPSC sheet induced the formation and outgrowth of neurites in neuronally differentiated SH-SY5Y neuroblastoma cells. These effects were enhanced when DPSC sheets were cultured with FGF2. The neurite-promoting effects were abolished when neurotrophic factors were inhibited, suggesting that they are needed for the positive effect of DPSC sheets on neuronal cell activity [57].

Recently, Chouaib et al. evidenced that DPSC-CM enhancement of neurite outgrowth in sensory neurons is concentration dependent. The authors also found that 48 h of DPSCs conditioning was the best option to obtain CM with efficient activity, while extending the conditioning time did not improve the effects of DPSC-CM. Interestingly, the frozen storage did not influence experimental outcomes. The CM contained some factors known for their role in neurogenesis and neuroprotection but also in angiogenesis and osteogenesis. Moreover, the conditioning of DPSCs with the B-27 supplement enhanced the neuroregenerative effects of their secretome, inducing a change of its composition in growth factors. In particular, CM was more efficacious when B-27 was added to DPSCs before conditioning [58].

CM from DPSCs enhanced neuritogenesis and exerted a chemoattractant effect also on neural stem cells (NSCs). The priming of DPSCs with leukocyte- and platelet-rich fibrin (L-PRF) increased BDNF secretion, but exerted no additional effects on the paracrine-mediated repair mechanisms [59].

DPSC-derived CM was also shown to be able to protect and regenerate isolated primary trigeminal ganglion neuronal cells (TGNC). Indeed, CM enhanced TGNC survival associated with extensive neurite outgrowth and branching. In parallel, DPSC-CM significantly upregulated NeuN, βIII-tubulin, and synapsin-I neuronal marker expression as well as TRPV1. Interestingly, DPSC-CM contained NGF, BDNF, NT-3, and GDNF [60].

G-CSF-mobilized DPSCs expressed higher neurotrophic factors compared to basal DPSCs and their secretome showed an enhanced neurite extension potential. Indeed, mobilized DPSC CM had a greater effect on neurite outgrowth in TGW cells [61]. Previously, it was demonstrated that CM from mobilized DPSC enhanced proliferation and migratory activity of neuronal Schwann RT4-D6P2T cells [62].

Interestingly, CM from SHEDs and DPSCs was shown to be able to promote the regeneration of cerebral granular neurons inhibiting axon growth inhibitors signals by paracrine mechanisms [63].

The DPSCs secretome also shows superior effects compared to other MSCs. Kumar et al. demonstrated that the secretome derived from DPSCs, SCAPs and DFSCs induced neural differentiation in IMR-32 cells, a preneuroblastic cell line, in a more efficient manner compared to BMSCs. In particular, neurite length was higher when IMR-32 cells were treated with the DPSC secretome. The DPSC secretome contained GCSF, IFN-γ, and TGF-β, which may promote neural differentiation [64].

DPSCs, BMSCs and AMSCs promoted an increase in the survival of co-cultured retinal ganglion cells. In particular, the increase in survival was enhanced in DPSC-treated retinal cultures. Interestingly, coculture with DPSC induced a significant increase in both the number of neurite-bearing retinal ganglion cells and neurite length compared with cocultures with BMSCs and AMSCs. However, these effects were blocked using neurotrophic factor receptors Fc-receptor blockers. The different types of MSCs showed a different pattern of neurotrophic factor expression, and, specifically, DPSCs released higher levels of several growth factors such as NGF, BDNF, and VEGF compared with BMSCs and AMSCs. In particular, VGF may mediate the neuroprotective effects of DPSCs [65].

CM-DPSCs showed protective effects on oxygen-glucose deprivation (OGD)-induced cytotoxicity in astrocytes in a dose-dependent manner. Specifically, both pre- and posttreatment with CM-DPSCs, but also CM-BMSCs, attenuated OGD-induced glial fibrillary acidic protein (GFAP), nestin, and musashi-1 expression in astrocytes. The treatment with CM also blocked OGD-induced reactive oxygen species (ROS) production and IL-1β upregulation. Interestingly, CM-DPSCs confer superior cytoprotection against cell death compared with BMSCs [66].

Venugopal et al. compared the neuroprotective potential of EXOs, CM or neuron–MSC-co-culture system against kainic-acid-induced excitotoxicity in vitro. Moreover, in order to identify the most adapt MSC type, EXOs and CM derived from DPSCs and BMSCs were tested. All three approaches showed neuroprotective potential thanks to the increase of growth factor expressions and the inhibition of apoptosis through the activation of PI3K-Bcl-2 pathway. It is important to note that EXOs demonstrated better anti-necrotic properties compared to neuron-MSC co-culture or CM. Regarding CM, only the fraction containing proteins in the range 3–10 kDa showed neuroprotection and rescued the neurons from excitotoxicity [67].

The secretome of DPSCs also showed beneficial effects in models of neurodegenerative diseases. Treatment with DPSC secretome reduced amyloid β (Aβ) cytotoxicity in an in vitro model of Alzheimer’s disease (AD), increasing cell viability and reducing apoptosis. DPSC secretome was shown to contain elevated levels of VEGF, Fractalkine, RANTES, monocyte chemoattractant protein-1 (MCP-1), and GMCSF compared to those of BMSCs and AMSCs. Interestingly, neprilysin, a protease able to degrade Aβ, was also found in the DPSC secretome. DPSC secretome proteolytically degrades Aβ_1–42_ in vitro, resulting in complete degradation after 12 h [68].

Early pre-symptomatic DPSC-CM administration improved neuromuscular junction innervation compared to vehicle-treated SOD1^G93A^ mice. The administration during late pre-symptomatic stages not only increased neuromuscular junction preservation, but also motor neuron survival in the spinal cord ventral horn. However, astrogliosis and microglia reactivity remained unaffected. Interestingly, the daily DPSC-CM treatment from symptom onset increased post-onset survival as well as overall lifespan [69].

DPSC-EXOs administration in a murine model of transient middle cerebral artery occlusion (tMCAO) injury reduced brain oedema, cerebral infarction, and neurological impairment. DPSC-EXOs inhibited the ischemia/reperfusion (I/R)-mediated expression of Toll-like receptor (TLR) 4, MyD88, and Nuclear Factor kappa-light-chain-enhancer of activated B cells (NF-κB). DPSC-EXOs also reduced the protein expression of pro-inflammatory cytokines IL-6, IL-1β and TNF-α, and cytoplasmic translocation of high mobility group box protein (HMGB) 1 in vivo but also in vitro in OGD/reperfusion (OGD/R)-induced BV2 cells. Thus, the results indicated that DPSC-EXOs may exert neuroprotection against cerebral I/R-induced neuroinflammation via the inhibition of the HMGB1/TLR4/MyD88/NF-κB signaling pathway [70].

DPSC-CM ameliorated aneurysmal subarachnoid hemorrhage (aSAH)-induced vasoconstriction and improved oxygenation in an injured brain. DPSC-CM administration also ameliorated cognitive and motor impairments. DPSC-CM administration decreased neuroinflammation as demonstrated by the reduction in the number of Iba1-positive cells. The major constituent of DPSC-CM was IGF-1. Antibody-mediated neutralization of IGF-1 moderately deteriorated the rescuing effect of DPSC-CM on microcirculation, Iba1-positive cells in the injured brain area, and the cognitive/motor impairments [71].

DPSC-CM administration ameliorated sciatic motor/sensory nerve conduction velocity, sciatic nerve blood flow, and intraepidermal nerve fiber density in the footpads of streptozotocin-induced diabetic rats. Furthermore, capillary density of the skeletal muscles increased while pro-inflammatory reactions in the sciatic nerves of diabetic rats were reduced [72]. Kanada et al. confirmed the positive effects of CM in sciatic nerve conduction velocity and sciatic nerve blood flow. Moreover, the treatment also increased muscle bundle size, vascular density in the skeletal muscles, and intraepidermal nerve fiber density in the diabetic rats. However, no differences were found between the results for DPSCs and DPSC-CM. These results suggested that the efficacy of DPSC and DPSC-CM administration were probably due to the secretome. In particular, DPSC-CM contained angiogenic factors such as VEGF-C, neurotrophic factors, such as BDNF, and immunomodulatory factors including IL-1β, IL-4, and TLR4 [73].

An overview of the studies presented in this paragraph is available in Table 2.

### 3.2. Stem Cells from Human Exfoliated Deciduous Teeth Secretome

Secretome of SHEDs was reported to modulate microglial cell activity. EVs derived from SHEDs inhibited lipopolysaccharide (LPS)-induced activation of NF-κB signaling pathway in human microglial cells. Moreover, EVs induced an upregulation of phagocytic activity in unpolarized cells, a slight decrease in M1 polarized cells, and a moderate increase in M2 polarized cells. EVs induced an immediate and sustained increase of glycolytic activity in M0, M1, and M2 polarized cells. Interestingly, EVs acted in an inverse dose-dependent manner [74]. EVs also induced a rapid increase in intracellular Ca^2+^ and ATP release in microglial cells. EVs were also able to promote microglial motility through P2X4 receptor/milk fat globule-epidermal growth factor-factor VIII (MFG-E8)-dependent mechanisms [75].

Different studies report beneficial effects of SHED CM in both in vitro and in vivo Parkinson’s disease (PD) models. Fujii et al. evidenced that dopaminergic-neuron-like cells induced from SHEDs were able to exert therapeutic benefits in a 6-hydroxy-dopamine (6-OHDA)-induced Parkinsonian rat model, improving neurological deficits and increasing dopamine (DA) levels more efficiently than undifferentiated SHEDs. However, paracrine effects may contribute to neuroprotection against 6-OHDA-induced neurodegeneration. Indeed, the CM obtained from differentiated SHEDs was able to protect primary neurons against 6-OHDA toxicity and accelerated neurite outgrowth in vitro [76]. Different doses of SHED-CM were tested in a PD model. The dose 10 µg/mL of SHED-CM did not restore motor ability, while 30 µg/mL of SHED-CM induced only mild improvements. Instead, 100 µg/mL of SHED-CM induced the maximal improvement of motor deficits in PD rats and a higher dose did not induce further improvement. SHED-CM increased tyrosine hydroxylase (TH) amounts and decreased synuclein levels in both the substantia nigra and striatum. In addition, SHED-CM treatment decreased both Iba-1 positive cells and CD4 levels in the same brain areas. The major constituents of SHED-CM included insulin-like growth factor binding protein-6 (IGFBP-6), tissue inhibitor of metalloproteinase (TIMP)-2, TIMP-1, and TGF-1. Moreover, bioinformatics analysis indicated that SHED-CM was able to promote neural regeneration. Indeed, RNA sequencing evidenced that SHED-CM administration shifted the gene expression profile to a pattern similar to that of control rats, upregulating genes that were involved in neurodevelopment and nerve regeneration. The major constituents of SHED-CM may participate in the molecular networks involved in cholinergic and serotoninergic synapses, calcium signaling pathways, and axon guidance [77].

EXOs and MVs derived from SHEDs have also been evaluated for their neuroprotective effects in PD models. EXOs, but not MVs, derived from SHEDs grown on laminin-coated three-dimensional alginate micro-carriers suppressed 6-OHDA-induced apoptosis in dopaminergic neurons. On the contrary, no protective effects were exerted by MVs or EXOs derived from SHEDs grown in standard culture conditions [78].

Instead, intranasal administration of EVs derived from SHEDs was shown to be effective in a rat model of PD, improving motor function in association with a normalization of TH expression in the striatum and substantia nigra [79].

The intranasal administration was also tested in an AD model, showing that SHED-CM improved cognitive function. SHED-CM reduced oxidative stress, shifted the M1-type pro-inflammatory microenvironment toward the M2-type anti-inflammatory and neuroprotective one, and increased neurotrophic factor levels. BMSCs-CM was less efficacious. It reduced oxidative stress and inflammation, but could not upregulate the expression of the anti-inflammatory M2 markers. Treatment with SHED-CM also suppressed glutamate-induced neuronal death in vitro [80].

SHED-CM was also able to improve disease scores and reduce demyelination, axonal injury, inflammatory cell infiltration, and proinflammatory cytokine expression in the spinal cord of experimental autoimmune encephalomyelitis (EAE) mice. These changes were associated with a change in the microglia/macrophage phenotype from M1 to M2. The treatment of EAE mice with the secreted ectodomain of sialic-acid-binding Ig-like lectin-9 (ED-Siglec-9), a major component of SHED-CM, resulted in similar effects compared to SHED-CM treatment, while ED-Siglec-9 depletion abolished the protective effects of SHED-CM. On the contrary, HGF depletion did not cause an inhibition of SHED-CM mediated protection, indicating that HGF had little effect on the efficacy of SHED-CM. SHED-CM inhibited the proliferation of myelin oligodendrocyte glycoprotein-specific CD4+ T cells, as well as their production of proinflammatory cytokines in vitro [81].

Matsubara et al. showed that SHED and SHED-CM administered into rat injured spinal cord during the acute postinjury period induced functional recovery. SHED-CM showed anti-inflammatory activity, reducing the levels of pro-inflammatory cytokines, and immunoregulatory action, inducing M2 anti-inflammatory macrophages. To identify factors responsible for the therapeutic effects of CMs, soluble factors present in SHED-CM were characterized. A total of 79 proteins were identified, some of them known to be involved in neuroregenerative processes, with anti-apoptotic, anti-inflammatory and axonal elongation properties. In particular, MCP-1 and ED-Siglec-9 may be involved in M2-like macrophage differentiation. Indeed, depleting these factors from the SHED-CM reduced CM’s ability to induce M2-like macrophages and to promote functional recovery after SCI. Interestingly, the administration of BMSC-CM induced no or only slight M2-like cell differentiation and did not induce recovery such as SHED-CM [82].

In agreement with the previous study, the treatment with SHED-CM loaded on a collagen hydrogel, used as a delivery system, induced functional recovery in SCI rats, as demonstrated by improvement in scores evaluated through Basso, Beattie, and Bresnahan scoring, inclined plane, cold allodynia, and beam walk tests [83]. The treatment with SHED-CM loaded on a collagen hydrogel also increased the volume of preserved white and gray matter and the total number of neurons and oligodendrocytes in a rat SCI model. On the contrary, lesion volume and lesion length decreased. However, in this study SHED-CM alone exerted no protection. The authors suggested that this may be due to the rapid diffusion of SHED-CM, and thus collagen hydrogel may act as an efficient releasing system [84].

A single intravenous injection of SHED-CM also reversed the mechanical allodynia induced by spinal nerve transection, suppressed microglia and astrocytes activation, and decreased the numbers of neurons positive for the neuronal injury marker activating transcription factor 3 (ATF3) and macrophage accumulation. In particular, the SHED-CM fraction with a molecular weight between 30 and 50 kDa reversed the pain, suggesting that protein components with molecular mass in the range 30–50 kDa were responsible for the reported neuroprotection [85].

The implantation of a collagen sponge enriched with the serum-free CM from SHED into the nerve gap formed by rat facial nerves transection restored the neurological function. On the contrary, CM depleted of MCP-1 and ED-Siglec-9, which are anti-inflammatory M2 macrophage inducers, did not restore neurological function. Notably, MCP-1 and ED-Siglec-9 induced the polarization of M2 macrophages in vitro and in vivo. Thus, the results indicated that MCP-1/ED-Siglec-9 participated in peripheral nerve regeneration inducing M2 macrophage [86].

SHED-CM treatment increased proliferation, migration, and the expression of neuron-, ECM-, and angiogenesis-related genes in Schwann cells. Moreover, SHED-CM stimulated neurite outgrowth of dorsal root ganglia and increased cell viability. In vivo, axon regeneration and myelination were higher in the SHED-CM group after nerve transection surgery. Motor function improved while muscle atrophy was reduced in the SHED-CM group. Thus, SHEDs may secrete various trophic factors that enhance peripheral nerve regeneration through multiple mechanisms. Specifically, SHED-CM contained NGF, BDNF, NT-3, GDNF, ciliary neurotrophic factor (CNTF), VEGF, and HGF [87].

The administration of SHED-EXOs improved rat motor functional recovery and reduced cortical lesion in rats with traumatic brain injury. SHED-EXOs can exert these effects, reducing neuroinflammation shifting microglia polarization [88].

SHED-CM induced an improvement of motor disability and reduced infarct volume after permanent MCAO. The SHED-CM treated group showed increased levels of doublecortin, neurofilament H, neuronal nuclei and rat endothelial cell antigen in the peri-infarct area. Interestingly, SHED-CM induced the migration and differentiation of endogenous neuronal progenitor cells (NPC), vasculogenesis and improved ischemic brain injury [89].

Intracerebral administration of SHED-CM in hypoxia-ischemia injured mice improved neurological function, survival rate, and neuropathological score [90].

CM obtained from SHEDs, and specifically only the fraction of <6 kDa, promoted neurite outgrowth of DRG neurons. Moreover, SHED-CM prevented the decline in sensory nerve conduction velocities in diabetic mice and ameliorated the capillary number-to-muscle fiber ratio and capillary blood flow [91].

In an animal model of superior laryngeal nerve injury, the systemic administration of SHED-CM induced functional recovery, increasing the degree of myelination and promoted axonal regeneration shifting macrophages toward the M2 phenotype [92].

An overview of the studies presented in this paragraph is available in Table 3.

### 3.3. Periodontal Ligament Stem Cell Secretome

CM obtained from PDLSCs of relapsing remitting multiple sclerosis (RR-MS) patients showed anti-inflammatory and antiapoptotic effects in NSC-34 mouse motor neurons stimulated with the medium of LPS treated RAW 264.7 macrophages. Indeed, CM treatment reduced TLR4 and NF-κB levels, together with pro-inflammatory cytokines. On the contrary, IL-10 increased in association with neuroprotective markers such as nestin, neurofilament 70, NGF, and GAP43. Interestingly, the neuroprotective effects of EVs may be due to their content in IL-10 and TGF-β [93].

The effects of PDLSCs-CM obtained from healthy donors and RR-MS patients were also evaluated in phorbol-12-myristate-13-acetate (PMA) differentiated THP-1, used as a model of microglia, and in undifferentiated and differentiated MO3.13 cells, used as models of progenitor cells and oligodendrocytes, respectively, treated with *Porphyromonas gingivalis* LPS. Treatment with both CM reduced the LPS-induced increase in TNFα, IL-1β and IL-6 levels and reduced TLR-4 in THP-1 cells [94].

PDLSCs-CM and purified EXOs/MVs (PDLSCs-EMVs) obtained from RR-MS patients and healthy donors exerted protective effects in EAE mice. In particular, PDLSCs-CM and PDLSCs-EMVs improved disease scores, restoring tissue integrity and remyelination in the spinal cord. PDLSCs-CM and PDLSCs-EMVs exerted anti-inflammatory effects both in spinal cord and spleen, as demonstrated by the reduction of pro-inflammatory cytokines and the induction of IL-10. In parallel, apoptosis was also inhibited. The anti-inflammatory effect of CM or EMVs might be due to the presence of the immunomodulatory cytokines IL-10 and TGF-β [95]. Moreover, PDLSCs-CM and EMVs obtained from RR-MS patients inhibited NALP3 inflammasome activation and reduced TLR-4 and NF-κB levels in EAE mice. The immunomodulatory factors IL-10, TGF-β, and SDF-1α included in the CM may be responsible for the immunosuppressive role of PDLSCs-CM and EMVs in EAE [96].

Interestingly, CM obtained from PDLSCs cultured under hypoxic conditions was efficacious in ameliorating clinical and histologic disease scores in EAE mice. In particular, this treatment reduced inflammatory cell infiltration and increased remyelination in the spinal cord. In particular, hypoxic CM administration increased the anti-inflammatory cytokine IL-37 in association with the reduction of proinflammatory cytokines. Moreover, oxidative stress and apoptosis were also inhibited, while BDNF increased. CM treatment was also able to regulate autophagy through the activation of the PI3K/Akt/mTOR pathway. Furthermore, in in vitro scratch injury exposed NSC-34 motor neurons, the hypoxia CM was able to modulate inflammation, oxidative stress, and apoptosis. Interestingly, hypoxia CM contained NT-3, IL-10, and TGF-β that may explain its neuroprotective effects [97].

An overview of the studies presented in this paragraph is available in Table 4.

### 3.4. Other Dental-Derived MSCs

Neuroprotective effects of CM obtained by GMSCs were evaluated in mechanically injured murine motor-neuron-like NSC-34 cells. CM treatment reduced the scratch injury-induced apoptosis and oxidative stress. Moreover, CM reduced TNF-α while increasing the levels of the anti-inflammatory cytokine IL-10. Interestingly, CM treatment upregulated BDNF and NT-3. CM was shown to contain NGF, NT-3, IL-10, and TGF-β, which may explain the neuroprotective effects [98].

EVs from GMSCs were tested for peripheral nerve regeneration in a crush-injured sciatic nerve mouse model. In vivo, the transplantation of Gelfoam embedded with GMSC-derived EVs at the crush injury site induced functional recovery and axonal regeneration in a similar way compared to the direct transplantation of GMSCs. In particular, EVs promoted proliferation and migration of Schwann cells and upregulated the protein expressions of c-JUN, Notch1, GFAP, and SOX2 genes associated with dedifferentiation or repair phenotype of Schwann cells. Also in vitro, EVs promoted the expression of Schwann cell dedifferentiation/repair genes [99]. A positive effect on Schwann cell proliferation was also reported for EXOs from GMSCs, which also promoted DRG axon growth in vitro. Moreover, the effects of GMSCs EXOs combined with biodegradable chitin conduits on peripheral nerve regeneration were evaluated. In vivo, in a rat sciatic nerve defect model, chitin conduit combined with EXOs increased the number and diameter of nerve fibers and promoted myelin formation. In parallel, nerve conduction also improved. Moreover, muscle function and motor function were ameliorated [100].

CM from SCAPs, DPSCs, and PDLSCs were tested to evaluate their capacity in inducing neurite outgrowth. With this aim, differentiated neuroblastoma SH-SY5Y cells were incubated with the different CM. The CM were shown to be able to increase the percentage of cells producing neurites and the total neurite outgrowth length. Interestingly, the length of the longest neurite per neuron was increased in a significant manner only with SCAP CM, and the neutralization of the secreted BDNF inhibited neurite outgrowth, indicating its importance in this process [101]. CM released from SCAPs showed also a greater neurogenic inductive effect on DPSCs compared to BMSCs-CM. Indeed, when DPSCs were cultured in medium for neural stem cell growth, the levels of neurogenic markers increased with the addition of SCAPs-CM. On the contrary, neuronal marker expression was reduced, while that of neurotrophic marker increased, when BMSCs-CM was added. Cell proliferation was not influenced by SCAPs-CM [102].

Oral mucosa stem cells (OMSCs) were differentiated into cells showing an astrocyte-like morphology and expressed characteristic astrocyte markers. The CM obtained by differentiated OMSCs increased the cell viability of motor neurons cultured in hypoxic conditions or exposed to hydrogen peroxide in vitro [103].

An overview of the studies presented in this paragraph is available in Table 5.

## 4. Translation of Secretome Application from the Preclinical Models into Clinical Use

The application of the secretome for a cell-free therapy may present some advantages compared to the use of MSCs. The main advantages of the use of the secretome instead of stem cell therapy are represented by the low immunogenicity and easier production, handling, and storage of the secretome [104]. Then, this therapy can overcome the risks linked to cell therapy, such as tumorgenicity, antigenicity, host rejection, and infections. Secretome handling can be easier compared to cells, given that it can be concentrated, frozen, and that it does not require liquid nitrogen storage and cell culture facilities, making its transfer also easier [105,106]. Moreover, secretome production is more economical and mass production under controlled laboratory conditions is also possible [107]. However, the period of survival of MSCs after transplantation is not clear, given that some data seem to indicate a limited survival [23].

Thus, therapeutic strategies based on secretome application could be helpful in regenerative medicine, based on their content in bioactive molecules, including proteins, mRNAs but also non-coding RNA, that can be useful to induce repair mechanism in the injured tissues. However, before translation into clinical applications, several points need to be clarified. In particular, it is necessary to better define the secretome composition, dosage, frequency, and route of administration. In this regard, it is also necessary to develop standardized manufacturing protocols with good manufacturing practice for the development of new pharmaceuticals based on cell-free products [105]. Indeed, for applications into clinical practice, the secretome should be presented in a standardized and easy to handle formulation. The secretome indeed may present variations on the basis of the subjects, cells, and tissues of origin [30]. For this reason, to standardize the secretome production it is necessary to define culture medium and supplements, culture duration, and culture conditions [106]. In this context, big data studies that evaluate proteome, transcriptome and non-coding RNA profile may provide assistance for secretome characterization. A list of studies analyzing transcriptome, non-coding RNA, and proteome profiling is available in Table 6.

However, secretome delivery also needs to address the concern of the rapid diffusion and clearance of secretome from the tissue repair site. Moreover, secretome stability and the stability of its growth factors and miRNAs need to be maintained in physiological conditions for all the delivery duration. In this context, different biomaterials have been developed and optimized in order to improve the delivery efficiency of MSCs secretome, with the advantages of a prolonged release duration, protection against degradation, and enhancing the therapeutic capacity [108]. Another option to improve the long-lasting stability of formulations is a freeze-drying process, which is used for different biological products [109,110].

## 5. Conclusions

Dental MSCs, given their origin from neural crest, have been shown to possess a prominent neuroregenerative potential. Dental MSC-derived secretome also shows the same enhanced neuroprotective and neuroregenerative properties. Both CM and EVs contain neurotrophins and molecules with a neuroprotective action, even at higher levels compared to other MSCs. The studies evaluated in this review highlighted that both CM and EVs stimulated neurite outgrowth and showed neuroprotective effects in preclinical models of neurological diseases and neuronal damage. In particular, the secretomes of DPSCs and SHEDs were the most studied, but different studies also highlighted the neuroprotective effects of PDLSC and GMSC secretomes. Interestingly, some studies also suggested the superiority of the secretome obtained from dental MSCs compared to other MSCs sources, such as BMSCs and AMSCs, for neuroprotection. In conclusion, the secretome derived from dental MSCs seems promising for its application in the neuroregenerative field and may be useful to develop new neuroprotective treatments.

## Figures and Tables

**Table 1:** Overview of the main factors found in the dental MSC secretome.

MSCs	Secretome	Contained Factors	Comparison with Other MSC Secretomes	Ref.
SCAPs	CM	2046 proteins, included chemokines, angiogenic, immunomodulatory, antiapoptotic, and neuroprotective factors, ECM proteins	151 proteins were different by at least twofold compared to BMSCs. SCAPs CM: ↑ proteins involved in metabolic processes, transcription, chemokines and neurotrophins. ↓ proteins linked to biological adhesion, developmental processes, immune function, ECM and pro-angiogenic factors	[31]
DPSCs	CM	Ang-2, EGF, Endoglin, Endothelin-1, Eotaxin-1, FGF-1, FGF-2, Flt-3L, Follistatin, G-CSF, GM-CSF, GRO pan, HB-EGF, HGF, IFNα2, IFNγ, IL-12(p40), IL-12(p70), IL-13, IL-15, IL-1B, IL-5, IL-8, IL-9, IP-10, Leptin, MCP-1, MCP-3, PDGF-AA, PDGF-BB, PLGF, RANTES, TGF-α, TGF-β1, TGF-β2, TGF-β3, TNFα, TNFβ, VEGF-A, VEGF-C, VEGF-D	Differences compared to UC-MSCs at different time points: Eotaxin-1, FGF-2, Fractalkine, GRO pan, IFNα2, IL-1α, IL-6, MCP-1, MCP-3, PDGF-BB, RANTES, TGF-β1, VEGF-A, VEGF-C	[32]
DPSCs	CM	IGF-1, IL10, IGFBP-6, NT-3, BMP-4, MIP-1δ, NAP-2, TGF-β3, TGF-β1, MIP-3α, TNF-α, TNF-β, ICAM-1, NT-4, I-TAC, TARC, Axl, THPO, TECK, Acrp-30, ICAM-3, EGFR, AgRP, XCL-1, MIF	Upregulated in DACCs: IGF-1, IL10, IGFBP-6 Downregulated in DACCs: NT-3, BMP-4, MIP-1δ, NAP-2, TGF-β3, TGF-β1, MIP-3α, TNF-α, TNF-β, ICAM-1, NT-4, I-TAC, TARC, Axl, THPO, TECK, Acrp-30, ICAM-3, EGFR, AgRP, XCL-1, MIF	[34]
PDLSCs	CM	99 proteins, including matrix proteins, enzymes, growth factors, cytokines, and angiogenic factors	-	[35]
DPSCs, SCAPs and DFSCs	CM	Osteogenic lineage related proteins were more in Dental MSC secretome	Dental MSCs showed more psteogenic protein compared to BMSCs	[36]
SHEDs	CM	FGF-2, IL-10, PDGF, SDF-1, Ang-1, TGF-β3, HGF, INF-γ, VEGF, and IL-6	↑ TGF-β3 and angiopoietin-1 in BMSCs, HGF and IFN-γ in SHED, VEGF in WJ-MSC. PDGF-A, IL-10, FGF-2, and SDF-1 were similar in all MSCs	[37]
Permanent and deciduous-teeth PDLSCs	CM	76 proteins in permanent-PDLSCs CM, 20 in deciduous-PDLSCs CM, and 19 in both samples; permanent-PDLSCs CM: proteins involved in cell growth, cell communication, and signal transduction, higher levels of NT-3 and NT-4 and angiogenesis-related cytokines such as EGF and IGF-1. Deciduous-PDLSCs CM: proteins involved in regulation of the cell cycle, cytokines involved in immune response and in tissue degradation and catalytic activities, including MMP1, PSMA1, and CUL7	-	[38]
GMSCs	EVs	Transcripts for growth factors such as TGF-β, FGF, VEGF, GDNF family ligands and neurotrophins, such as NGF, BDNF, NT-3 and NT-4, ILs and members of the Wnt family	-	[41]
PDLSCs	EVs	Contained non-coding RNA: antisense RNA, long non-coding RNA, miRNAs (MIR24-2, MIR142, MIR335, MIR490, and MIR296)	-	[42]
SCAPs	EXOs	593 piRNAs	920 piRNAs in BMSC-EXOs. 21 piRNAs were differentially expressed	[43]
DPSCs	CM	CM obtained in normoxic conditions: ↑ molecules with anti-inflammatory, tissue repair and regenerative properties compared to hypoxic CM	-	[44]
SHEDs, young DPSCs, old DPSCs	CM	IL-4, IL-2, CXCL10, IL-1B, TNF-A, CCL2, IL-17A, IL-6, IL-10, IFN-γ, IL-12P17, CXCL8, TGF-β1, ANG-2, EGF, EPO, BFGF, G-CSF, GM-CSF, HGF, M-CSF, PDGF-AA, PDGF-BB, SCF, TGF-α, VEGF SHEDs and young DPSCs: ↑ growth factors, ↓ pro-inflammatory cytokines	-	[47]
DPSCs treated with THSG	CM	Treatment increased: AKT2, persephin, NGFR, PTHrP, maspin, leptin, STAT3, YES1, MMP-13, FGF-5, HER3, FGF-16, IGF-BP1, LH, myostatin, HDAC1, SDF-1β, MDC, MCP-4, L-selectin, TNF-α, STAT6, β-2-MICROGLOBULIN, APRIL, eotaxin-3, MCP-1, LIGHT, galectin 3, LD78β, MIP-1β, granzyme B, LEC. Treatment reduced: TSH, EGF, CTGFL, HGH, FSH, vaspin, PDGF-AA, GM-CSF, KGF, FGF-acidic, FAK, GDF-3, TGF-β2, IGF-I, MMP-2, STAT-1, TIMP-1, eotaxin, MMP-23, IL-12, galectin-1, ena-78, IL-15, IL-4, IL-22, IL-6, IL-13, IL-1β	-	[49]
FGF-2-modified GMSCs	CM	↑ VEGF-A, FGF-2, TGF-β.	-	[50]
SHEDs treated with Ascorbic acid	CM	Treatment ↑ VEGF, TGF-α, SCF, TGF-β, IGF-1, HGF, bFGF, Ang-1, EGF, Ang-2, TNF-α, IL-10, IL-6, IL-17A, NO, IDO, SDF-1, PGE-2. Treatment ↓ CCL2, TGF-β1	-	[51]
Undifferentiated PDLSCs or PDLSCs exposed to osteogenic differentiation medium	EVs	69–557 circRNAs and 2907–11,581 lncRNAs. After 5 and 7 days of exposure to differentiation medium: ↑ 3 circRNAs and 2 lncRNAs, ↓ 39 circRNAs and 5 lncRNAs	-	[52]
Undifferentiated PDLSCs or osteogenic differentiated PDLSCs	EXOS	72 upregulated miRNAs, 35 downregulated miRNAs after osteogenic induction	-	[53]

Ang, angiopoietin; BDNF, brain-derived neurotrophic factor; BMP, bone morphogenetic protein; BMSCs, bone marrow MSCs; circRNA, circular RNA; CM, conditioned medium; CUL7, cullin 7; CXCL, C-X-C motif chemokine ligand; DACCs, developing apical complex cells; DFSCs, dental follicle stem cells; DPSCs, dental pulp stem cells; ECM, extracellular matrix; EGF, epidermal growth factor; ECM, extracellular matrix; EVs, extracellular vesicles; EXOs, exosomes; FGF, fibroblast growth factor; G-CSF, granulocyte colony-stimulating factor; GDNF, glial-cell-derived neurotrophic factor; GM-CSF, granulocyte-macrophage colony-stimulating factor; GMSCs, gingival MSCs; HGF, hepatocyte growth factor; ICAM, Intercellular Adhesion Molecule; IDO, indoleamine 2,3-dioxygenase; IFN, interferon; IGF, insulin-like growth factor; IL, interleuchin; lncRNA, long noncoding RNA; MCP, Monocyte Chemoattractant Protein; miRNA, microRNA; MMP, matrix metalloproteinase; NGF, nerve growth factor; NT, neurotrophin; PDGF, platelet-derived growth factor; PDLSCs, periodontal ligament stem cells; piRNA, PIWI-interacting RNAs; PSMA1, Proteasome subunit, alpha type; SCAPs, stem cells from apical papilla; SDF, stromal cell–derived factor; SHEDs, stem cells from human exfoliated deciduous teeth; TGF, transforming growth factor; THSG, 2,3,5,4′-tetrahydroxystilbene-2-O-β-D-glucoside; TIMP, tissue inhibitor of metalloproteinase; TNF, Tumor Necrosis Factor; UC-MSCs, umbilical cord mesenchymal stem cells; VEGF, vascular endothelial growth factor ↑, increase/improvements; ↓, reduction.

**Table 2:** Overview of studies involving dental pulp stem cell secretome.

Secretome Type	Administration	Preclinical Model	Factors Contained in Secretome	Results	Ref.
CM	-	Dorsal root ganglion neurons; Schwann cell	-	↑ neurite outgrowth, Schwann cell viability and myelin formation	[54]
CM	-	PC12 cells	NGF, BDNF, GDNF and NT-3	↑ survival and neurite outgrowth	[55]
CM	-	SH-SY5Y cell line	-	↑ neurite outgrowth, neuronal markers and voltage-gated Ca^2+^ channels	[56]
CM obtained by DPSC sheet	-	Neuronally differentiated SH-SY5Y neuroblastoma cells	BDNF, GDNF, NT-3	↑ formation and outgrowth of neurites	[57]
CM with and without B-27 supplement	-	Primary sensory neurons	Only with B-27: GDF-15, SCF R, Insulin; Only without B-27: FGF-4, GH, and VEGF-D; In common: NT-3, PDGF-AA, HGF, IGFBP (1–6), EGFR, OPG, VEGF, BMP-7, FGF-7, and IGF-1.	↑ neurite outgrowth, B-27 supplement enhanced the effect	[58]
CM	-	Neural stem cells	-	↑ neuritogenesis	[59]
CM	-	Primary trigeminal ganglion neuronal cells	NGF, BDNF, NT-3 and GDNF	↑ survival, extensive neurite outgrowth and branching.	[60]
CM obtained by basal and G-CSF-mobilized DPSCs	-	TGW human neuroblastomacells	-	CM from mobilized DPSCs ↑ neurite extension	[61]
CM obtained by G-CSF-mobilized DPSCs	-	Neuronal Schwann RT4-D6P2T cells	-	↑ proliferation and migratory activity	[62]
CM from SHEDs and DPSCs	-	Cerebral granular neurons with axon growth inhibitors	-	↑ regeneration inhibiting axon growth inhibitors	[63]
CM from DPSCs, SCAPs and DFSCs	-	Preneuroblastic cell line IMR-32 cells	GCSF, IFN-γ, TGF-β, NGF, BDNF and NT-3	↑ neural differentiation. Neurite length was higher with DPSC CM.	[64]
DPSC, BMSC and AMSC coculture	-	Retinal ganglion cells	NGF, BDNF, NT-3, VEGF, GDNF, PDGF-AA	↑ survival. DPSCs released higher levels of NGF, BDNF and VEGF	[65]
CM	-	Astrocytes exposed to OGD	-	↑ protective effects. ↓ GFAP, nestin, and musashi-1 expression, ROS and IL-1β	[66]
EXOs, CM or neuron–MSC-co-culture from DPSCs or BMSCs	-	Hippocampal cell line (H3) exposed to kainic acid	-	↑ neuroprotection. CM fraction in the range 3–10 kDa showed neuroprotection	[67]
CM	-	Human neuroblastoma SH-SY5Y cells treated with A𝛽_1–42_	VEGF, RANTES, FRACTALKINE, FLT-3, GM-CSF, MCP-1, neprilysin	↓A𝛽 cytotoxicity and apoptosis. ↑ cell viability. DPSC secretome proteolytically degrade A𝛽_1–42_.	[68]
CM	Intraperitoneally; early pre-symptomatic stage: at postnatal day 35–47; late pre-symptomatic stages: postnatal day 70–91; at symptom onset through to end-stage	Transgenic mice B6SJL-Tg (SOD1G93A)1 Gur/J	-	↑ neuromuscular junction innervation; neuromuscular junction preservation, and motor neuron survival, lifespan	[69]
EXOs	Intravenous singular injection after reperfusion	C57BL/6 mice subjected to tMCAO injury followed by reperfusion.OGD/R induced BV2 cells	-	In vivo: ↓ brain oedema, cerebral infarction, neurological impairment and pro-inflammatory cytokines. In vitro: ↓ inflammation	[70]
CM	Intrathecal injection 10 min before aSAH	Aneurysmal subarachnoid hemorrhage induced in Wistar rats	IGF-1, TGF-β, TIMP1, TIMP2	↑ oxygenation of injured brain, cognitive and motor function. ↓ neuroinflammation	[71]
CM	Hindlimb skeletal muscles 8 weeks after streptozotocin injection	Sprague Dawley rats treated with Streptozotocin	-	↑ sciatic motor/sensory nerve conduction velocity, sciatic nerve blood flow and intraepidermal nerve fiber density	[72]
CM	In the unilateral hindlimb skeletal muscles	Sprague Dawley rats treated with Streptozotocin	VEGF-C, BDNF, IL-1 β, IL-4, TLR4 and others	↑ sciatic nerve conduction velocity, sciatic nerve blood flow.	[73]

Aβ, amyloid β; aSAH, aneurysmal subarachnoid hemorrhage; AMSCs, adipose tissue derived MSCs; BDNF, brain-derived neurotrophic factor; BMP, bone morphogenetic protein; BMSCs, bone marrow MSCs; CM, conditioned medium; DFSCs, dental follicle stem cells; DPSCs, dental pulp stem cells; EXOs, exosomes; FGF, fibroblast growth factor; GDNF, glial-cell-derived neurotrophic factor; GFAP, glial fibrillary acidic protein; G-CSF, granulocyte colony-stimulating factor; HGF, hepatocyte growth factor; IFN, interferon; IGF, insulin-like growth factor; IGFBP, insulin-like growth factor binding protein; IL, interleukin; MCP, Monocyte Chemoattractant Protein; MSCs, mesenchymal stem cells; NGF, nerve growth factor; NT, neurotrophin; OGD/R, oxygen-glucose deprivation–reperfusion; OPG, osteoprotegerin; PDGF, platelet-derived growth factor; ROS, reactive oxygen species; SCAPs, stem cells from apical papilla; SHEDs, stem cells from human exfoliated deciduous teeth; TGF, transforming growth factor; TIMP, tissue inhibitor of metalloproteinase; TLR, Toll like receptor; tMCAO, transient middle cerebral artery occlusion; VEGF, vascular endothelial growth factor; ↑, increase/improvements; ↓, reduction.

**Table 3:** Overview of studies involving SHED secretome.

Secretome Type	Administration	Preclinical Model	Factors Contained in Secretome	Results	Ref.
EVs	-	SV40 human microglial cell line treated with LPS or polarized toward M1 or M2	-	↓ NF-κB signaling ↑ glycolytic activity in M0, M1, and M2 cells	[74]
EVs	-	Human microglial cells	-	↑ intracellular Ca^2+^ and ATP release, motility through P2X4 receptor/milk fat globule-epidermal growth factor-factor VIII (MFG-E8)-dependent mechanisms	[75]
CM from DAergic-neuron-like differentiated and undifferentiated SHEDs	-	Cerebellar granule neurons treated with 6-OHDA	-	CM obtained from differentiated SHEDs protect primary neurons against 6-OHDA toxicity and accelerated neurite outgrowth	[76]
CM	Intravenous injection	Lewis rats treated with rotenone	IGFBP-6, TIMP-2, TIMP-1, TGF-β1, IGFBP-2, IGFBP-4, BMP-5	100 µg/mL of SHED-CM induced the maximal improvement of motor deficits ↑ tyrosine hydroxylase ↓ synuclein levels, Iba-1 positive cells and CD4 levels	[77]
EXOs and MVs from SHEDs grown in standard culture conditions or on laminin-coated three-dimensional alginate micro-carriers	-	Dopaminergic neurons differentiated from ReNcell VM human neural stem cells treated with 6-OHDA	-	EXOs, but not MVs, derived from SHEDs grown on laminin-coated three-dimensional alginate micro-carriers suppressed apoptosis	[78]
EVs	Intranasal	Male Wistar rats treated with 6-OHDA	-	↑ motor function	[79]
CM	Intranasally 24 h after i.c.v. injection of A𝛽_1−40_	Mice receiving an i.c.v. injection of A𝛽_1−40_ peptide	-	↑ cognitive function and neurotrophic factors. ↓ oxidative stress	[80]
CM	Intravenous at day 14 postimmunization (peak of EAE)	EAE induced C57BL/6J mice	ED–Siglec-9 and HGF	↓ demyelination and axonal injury, inflammatory cell infiltration and proinflammatory cytokine expression	[81]
CM	Intrathecally	Sprague Dawley rats with spinal cord contusion injury	79 proteins of which 28 involved in neuroregenerative processes; MCP-1 and ED-Siglec-9 may be involved in M2-like macrophage differentiation	↑ functional recovery ↓ pro-inflammatory cytokines	[82]
CM alone or loaded on collagen hydrogel	Intraspinal injection	Sprague Dawley rats subjected to SCI	-	CM on collagen hydrogel ↑ functional recovery	[83]
CM alone or loaded on collagen hydrogel	Intraspinal injection	Sprague Dawley rats subjected to SCI	-	CM on collagen hydrogel: ↑ volume of preserved white and gray matter and the total number of neurons and oligodendrocytes ↓ lesion volume and lesion length	[84]
CM	Intravenous administration 5 days after peripheral nerve injury	Male C57BL/6J mice subjected to peripheral nerve injury made by the transection of the L4 spinal nerve	-	↓ allodynia and activation of microglia and astrocytes	[85]
CM	CM loaded on the collagen sponge placed in the nerve gap	Female Sprague Dawley with rat facial nerves transection	MCP-1 and ED-Siglec-9	↑ neurological function	[86]
CM	Silicon conduits containing CM	Schwann cells and dorsal root ganglia; male Wistar/ST rats subjects to sciatic nerve transection	NGF, BDNF, NT-3, GDNF, CNTF, VEGF, and HGF	In vitro: ↑ proliferation, migration, and expression of neuron-, ECM-, and angiogenesis-related genes, neurite outgrowth of dorsal root ganglia and increased cell viability In vivo: ↑ axon regeneration, myelination, motor function. ↓ muscle atrophy	[87]
EXOs	-	BV-2 cells treated with LPS; Wistar rats subjected to TBI	-	↑ motor function	[88]
CM	Intranasally 3 days after MCAO	Sprague Dawley rats subjected to permanent MCAO	-	↓ motor disability and infarct volume	[89]
CM	Intracerebral administration 24 h after hypoxia-ischemia	ICR mice with hypoxia-ischemia brain injury	-	↑ neurological function and survival rate	[90]
CM	Injection into soleus muscles twice a week over a period of 4 weeks twelve weeks after the induction of diabetes	Dorsal root ganglion neurons; C57BL/6 mice treated with streptozotocin	NGF, BDNF, FGF2 and VEGF in fraction > 6 kDa	In vitro: ↑ neurite outgrowth In vivo: ↓ decline in sensory nerve conduction velocities	[91]
CM	Intravenous simultaneously with the superior laryngeal nerve damage	Male Wistar/ST rats subjected to superior laryngeal nerve damage	-	↑ functional recovery, myelination and axonal regeneration	[92]

6-OHDA, 6-hydroxy-dopamine; Aβ, amyloid β; BDNF, brain-derived neurotrophic factor; BMP, bone morphogenetic protein; CM, conditioned medium; CNTF, ciliary neurotrophic factor; EAE, experimental autoimmune encephalomyelitis; ECM, extracellular matrix; ED-Siglec-9, ectodomain of sialic-acid-binding Ig-like lectin-9; EXOs, exosomes; EVs, extracellular vesicles; FGF, fibroblast growth factor; GDNF, glial-cell-derived neurotrophic factor; HGF, hepatocyte growth factor; i.c.v., intracerebroventricular; IGFBP, insulin-like growth factor binding protein; LPS, lipopolysaccharide; MCP, Monocyte Chemoattractant Protein; MVs, microvesicles; NF-κB, Nuclear Factor kappa-light-chain-enhancer of activated B cells; NGF, nerve growth factor; NT, neurotrophin; OGD, oxygen-glucose deprivation; SHEDs, stem cells from human exfoliated deciduous teeth; MCAO, middle cerebral artery occlusion; SCI, spinal cord injury; TBI, traumatic brain injury; TGF, transforming growth factor; TIMP, tissue inhibitor of metalloproteinase; VEGF, vascular endothelial growth factor; ↑, increase/improvements; ↓, reduction.

**Table 4:** Overview of studies involving PDLSC secretome.

Secretome Type	Administration	Preclinical Model	Factors Contained in Secretome	Results	Ref.
CM obtained from PDLSCs of RR-MS patients	-	NSC-34 neurons treated with the medium of Lipopolysaccharide-stimulated RAW macrophage	IL-10, TGF-β	↓ inflammation and apoptosis ↑ IL-10 and neuroprotective markers	[93]
CM from PDLSCs of healthy subjects or RR-MS patients	-	PMA differentiated THP-1 cells, undifferentiated and PMA-differentiated MO3.13 cells treated with Porphyromonas gingivalis LPS	-	↓ TNFα, IL-1β and IL-6 levels	[94]
CM and EXOs/MVs (EMVs) from PDLSCs obtained from RR-MS patients and healthy donors	Intravenous at disease onset	EAE induced C57BL/6 mice	IL-10 and TGF-β	↑ remyelination of spinal cord ↓ inflammation in spinal cord and spleen and apoptosis	[95]
CM and EXOs/MVs (EMVs) from PDLSCs obtained from RR-MS patients	Intravenous at disease onset	EAE induced C57BL/6 mice	IL-10, SDF-1α, TGF-β, IL-15, MCP-1 and MIP-1α	↓ NALP3 inflammasome activation, TLR-4 and NF-κB	[96]
CM obtained from PDLSCs cultured in hypoxic conditions	Intravenous 14 days after EAE induction	EAE induced C57BL/6 mice; Scratch injured murine motor-neuron-like NSC-34 cells	NT3, IL-10 and TGF-β	In vivo: ↓ clinical and histologic disease score, inflammatory cell infiltration, oxidative stress and apoptosis↑ remyelination in spinal cord, IL-37, activation of the PI3K/Akt/mTOR pathway. In vitro: ↓ inflammation, oxidative stress, and apoptosis	[97]

CM, conditioned medium; EAE, experimental autoimmune encephalomyelitis; EMVs, EXOs/MVs; EXOs, exosomes; IL, interleukin; MCP, Monocyte Chemoattractant Protein; MIP, Macrophage Inflammatory Protein; MVs, microvesicles; NF-κB, Nuclear Factor kappa-light-chain-enhancer of activated B cells; NT, neurotrophin; PMA, Phorbol 12-myristate 13-acetate; PDLSCs, periodontal ligament stem cells; RR-MS, relapsing remitting multiple sclerosis; SDF-1α, stromal cell–derived factor 1α; TGF, transforming growth factor; TLR, Toll like receptor; TNF, Tumor Necrosis Factor; ↑, increase/improvements; ↓, reduction.

**Table 5:** Overview of studies involving other dental MSCs secretome.

MSCs	Secretome Type	Administration	Preclinical Model	Factors Contained in Secretome	Results	Ref.
GMSCs	CM	-	Scratch injured murine motor-neuron-like NSC-34 cells	NGF, NT-3, IL-10 and TGF-β	↓ apoptosis, oxidative stress, TNF-α; ↑ IL-10, BDNF and NT-3	[98]
GMSCs	EVs	Gelfoam mixed with EVs wrapped around the injury site	C57BL/6J mice subjected to crush injury of sciatic nerve; rat Schwann cell line RT4-D6P2T	-	In vivo: ↑ functional recovery and axonal regeneration, proliferation and migration of Schwann cells. In vitro: ↑ expression of Schwann cell dedifferentiation/repair phenotype-related genes	[99]
GMSCs	EXOs	Chitin conduit enriched with EXOs	DRG cell co-cultured with Schwann cell; SD rats subjected to sciatic nerve defect	-	In vitro: ↑ Schwann cell proliferation, DRG axon growth. In vivo: ↑ number and diameter of nerve fibers, myelin formation, nerve conduction, muscle function and motor function	[100]
SCAPs, DPSCs and PDLSCs	CM	-	Retinoic acid differentiated SH-SY5Y cells	BDNF, NGF, NT-3, GDNF, VEGF-A and angiopoietin-1	↑ the percentage of cells producing neurites and the total neurite outgrowth length	[101]
SCAPs	CM	-	DPSCs cultured in medium for neural stem cell growth	-	↑ neurogenic effect compared to BMSCs-CM, neurogenic markers	[102]
Astrocyte-like differentiated OMSCs	CM	-	Mouse motor neuron-like NSC-34 cells exposed to hypoxia or hydrogen peroxide	-	↑ cell viability	[103]

BDNF, brain-derived neurotrophic factor; BMSCs, bone marrow MSCs; CM, conditioned medium; DPSCs, dental pulp stem cells; DRG, dorsal root ganglion; EVs, extracellular vesicles; EXOs, exosomes; GDNF, glial-cell-derived neurotrophic factor; GMSCs, gingival MSCs; IL, interleukin; NGF, nerve growth factor; NT, Neurotrophin; OMSCs, oral mucosa stem cells; PDLSCs, periodontal ligament stem cells; SCAPs, stem cells from apical papilla; TGF, transforming growth factor; TNF, Tumor Necrosis Factor; VEGF, vascular endothelial growth factor; ↑, increase/improvements; ↓, reduction.

**Table 6:** Studies analyzing proteome, transcriptome, and non-coding RNA profiles of different dental MSCs.

MSCs Type	Secretome	Profile	Ref.
SCAPs	CM	Proteome	[31]
DPSCs	CM	Metabolomic and bioactive factors profiles	[32]
PDLSCs	CM	Proteome	[35]
DPSCs, SCAPs, DFSCs	CM	Proteome	[36]
PDLSCs	CM	Proteome	[38]
GMSCs	EVs	Transcriptome	[41]
PDLSCs	EVs	Non-coding RNA	[42]
SCAPs	EXOs	piRNA profile	[43]
PDLSCs	EVs	circRNA and lncRNA profile	[52]
PDLSCs	EXOs	miRNA profile	[53]

circRNAs, circular RNA; CM, conditioned medium; DFSCs, dental follicle stem cells; DPSCs, dental pulp stem cells; EVs, extracellular vesicles; EXOs, exosomes; GMSCs, gingival MSCs; lncRNAs, long non-coding RNAs; miRNA, microRNA; PDLSCs, periodontal ligament stem cells; piRNA, PIWI-interacting RNAs; SCAPs, stem cells from apical papilla.
